# Vitamin-mineral supplements do not guarantee the minimum recommendations and may imply risks of mercury poisoning in dogs and cats

**DOI:** 10.1371/journal.pone.0250738

**Published:** 2021-04-26

**Authors:** Rafael Vessecchi Amorim Zafalon, Mariana Pamplona Perini, Thiago Henrique Annibale Vendramini, Vivian Pedrinelli, Mariana Fragoso Rentas, Isabela Benelli Morilha, Lucas Ben Fiuza Henríquez, Renata Maria Consentino Conti, Marcio Antonio Brunetto

**Affiliations:** 1 Department of Animal Nutrition and Production, Pet Nutrology Research Center (CEPEN-PET), School of Veterinary Medicine and Animal Science, University of Sao Paulo (USP), Pirassununga, Brazil; 2 Veterinary Nutrology Service (VNS), Veterinary Teaching Hospital, School of Veterinary Medicine and Animal Science, University of Sao Paulo (USP), São Paulo, Brazil; University of Life Sciences in Lublin, POLAND

## Abstract

Given the importance of using the vitamin-mineral supplements to guarantee the minimum nutritional recommendations for pets in homemade foods, and hypothesizing that these products may contribute to toxic metals contamination, the present study aimed to determine the concentrations of essential minerals and toxic metals in vitamin-mineral supplements available in the Brazilian market and calculate if the amount recommended by the manufacturer guarantees the minimum recommendations of NRC (2006) and FEDIAF (2020), as well as calculating the amount of toxic metals that animals would consume, according to the amounts recommended by the manufacturer. Seven vitamin-mineral supplements were analyzed. The determination of essential minerals and toxic metals was performed using ICP-OES. Comparisons were made with the minimum recommendations for essential minerals, and with the maximum tolerated levels of toxic metals established by the FDA (2011), descriptively. Most of the vitamin-mineral supplements, in the quantities recommended by the manufacturers, do not guarantee the minimum recommendations of NCR (2006) and FEDIAF (2020) for the following elements: calcium, potassium, magnesium, sodium, phosphorus, selenium, and zinc. Only one supplement had detectable selenium concentrations. Three supplements provided more than 0.02mg of mercury/kg of body weight, the safe upper limit used to establish the maximum tolerated level of this element. It is concluded that most vitamin-mineral supplements do not meet the minimum recommendations for most essential minerals and, if formulated by untrained professionals, even with supplementation, homemade foods may still be nutritionally deficient. Furthermore, some vitamin-mineral supplements analyzed may imply risks of mercury poisoning in pets.

## Introduction

Nowadays, there is ample growth in the search for homemade food for dogs and cats by their owners. This interest in homemade foods can occur due to several factors, such as difficulty in understanding the labels of processed products, concern about the presence of preservatives and dyes, satisfaction in preparing food for their pets, and greater palatability [1–4].

It should be noted that the consumption of more than 10% of daily calories from homemade diets is considered a nutritional risk factor [5]. According to Parr and Remillard [6], the use of supplementation of vitamins and minerals in the formulation of homemade diets is mandatory, since the requirement for these nutrients cannot be met only with the use of common ingredients. This occurs mainly because pet owners, as well as professionals not specialized in nutrition, often apply knowledge about human nutrition in the preparation of homemade diets for their pets, without taking into account the great difference in nutritional needs between species.

According to AAFCO [7], the vitamin- mineral supplements are basic blends intended to be mixed with other ingredients such as meat, carbohydrates and lipid components to provide a complete meal for pets. Therefore, it is not mandatory that they meet the minimum recommended vitamin and mineral requirement. However, some published studies [4, 8–10] show that most homemade diet recipes for dogs and cats present on websites [11–36] and books [37–46] have at least one nutritional deficiency when compared to the recommendations of guidelines like NRC [47] and FEDIAF [48]. Thus, it would be safer if the recommended dose on the label of these supplements reached the minimum requirements of these guidelines, bringing greater security to owners who are interested in providing this type of diet to their animals.

It has already been demonstrated by Pedrinelli et al. [4pedrinelli] that there may be high concentrations of toxic metals in homemade diets for dogs and cats, mainly of the elements: mercury, lead, cobalt, uranium, and vanadium. It has not been clarified from which ingredients these high concentrations originated, but it is believed that, among other ingredients, vitamin-mineral supplements may contribute to the contamination of the final product. In the study conducted by Pedrinelli at al. [4], there was a moderate correlation between the vitamin-mineral supplement used and cobalt concentrations.

Given the absence of research in the literature, the present study aimed to analyze essential minerals and toxic metals in vitamin-mineral supplements present in the Brazilian market, through which it can be evaluated whether these products guarantee the minimum recommendation of minerals for dogs and cats, as well as whether these products can contribute to the contamination of toxic metals in the final product.

## Material and methods

A search was conducted to gather all vitamin-mineral supplements registered in Brazil. First, the terms "nutritional supplement", "vitamin supplement", and "mineral supplement" were used to search for all products of this category in the database of Brazil’s Ministry of Agriculture. Then, a telephone survey of the country’s main pet food distributors was carried out to confirm whether these products were available. Based on this research, vitamin-mineral supplements were purchased at the main pet shop chains located in the city of São Paulo—Brazil. As a criterion for the acquisition of supplements, all products that had essential minerals in their composition and clearly recommended for supplementation of homemade diets were includeed, and one package of each supplement was purchased. Supplements that had only essential amino acids or vitamins but did not have minerals in their composition were not included. The commercial names of the analyzed supplements are: food dog maintenance, food dog growth, food dog senior, food dog minerals, complet, aminomix and food cat. The vitamin-mineral supplements were randomly numbered from 1 to 7, without identification, to maintain manufacturers privacy.

### Sample preparation

Sample preparation was performed by closed vessel digestion in a microwave oven (Multiwave GO, Anton Paar, Austria) according to the methodology described by Da Costa et al. [49] and Pedrinelli et al. [4]. The procedure was performed in duplicate. The preparation of samples for microwave digestion was carried out in the Analysis Laboratory of Biorigin Brasil (Lençóis Paulista, Sao Paulo, Brazil).

### Determination of essential minerals and toxic metals

The determination of all elements was performed by optical emission spectrometry with inductively coupled plasma [ICP-OES (ICPE-9000), Shimadzu do Brazil, Barueri, Brazil] at the Multiuser Laboratory of Animal Nutrition and Bromatology of the Department of Animal Nutrition and Production at the School of Veterinary Medicine and Animal Science of the University of Sao Paulo (Pirassununga, Sao Paulo, Brazil). To determine the elements antimony (Sb), arsenic (As), mercury (Hg), and selenium (Se), a hydride generator (hydride ICP, Elemental Scientific, Omaha, USA) was used coupled to the ICP-OES, according to the methodology described by Pedrinelli et al. [4]. It was not possible to determine chloride and iodine, as the digestion methodology employed did not allow it, due to the high ionization energy of these elements. Toxic metals determined were: aluminum (Al), Sb, As, boron (B), barium (Ba), beryllium (Be), cadmium (Cd), lead (Pb), cobalt (Co), chromium (Cr), tin (Sn), Hg, nickel (Ni), uranium (U), and vanadium (V).

It was stated on the label of each supplement analyzed the recommendation of the quantity to be supplied of the product for animals of different body weights (BW), or simply the quantity to be supplied per kg of BW of the animal. According to this information and the ICP-OES analyses results, the amount that each supplement provides of essential minerals per kg of metabolic weight (MW) of the animals and the amount of toxic metals per kg of BW was calculated. Metabolic weight was considered as BW^0.75^ for dogs and BW^0.67^ for cats [48]. In addition, based on the quantity to be added for each kg of prepared unconventional food declared on the labels, the amount of toxic metals was estimated per kg of dry matter of the homemade food, using as a standard humidity of 60% for the homemade food. The maximum tolerable level (MTL) established by the Food and Drug Administration (FDA) [50] for toxic metals was considered to evaluate if the concentrations of toxic metals in the final product were above the MTL, not considering the concentrations present in the ingredients of homemade foods.

### Statistical analysis

Comparisons were made between the quantities supplied of essential minerals per kg of MW and the recommendations of the National Research Council (NRC) [47] and the *Fédération Européenne de l’Industrie des Aliments pour Animaux Familiers* (FEDIAF) [48], descriptively. Regarding toxic metals, comparisons were made between the concentrations (in mg/kg) of these elements in the final product (homemade food) and the maximum tolerable level established by the FDA [50], also descriptively.

## Results

A total of seven vitamin-mineral supplements from three different manufacturers were purchased, of which two were indicated for both dogs and cats (Supplements 1 and 2), one for cats (Supplement 3), and four were indicated for dogs (Supplements 4–7), one of them exclusive for growing dogs (Supplement 7). Supplement 1 was purchased directly through the manufacturer’s website, as it was the only place it was available. All supplements were manufactured in Brazil.

**Fig 1:**
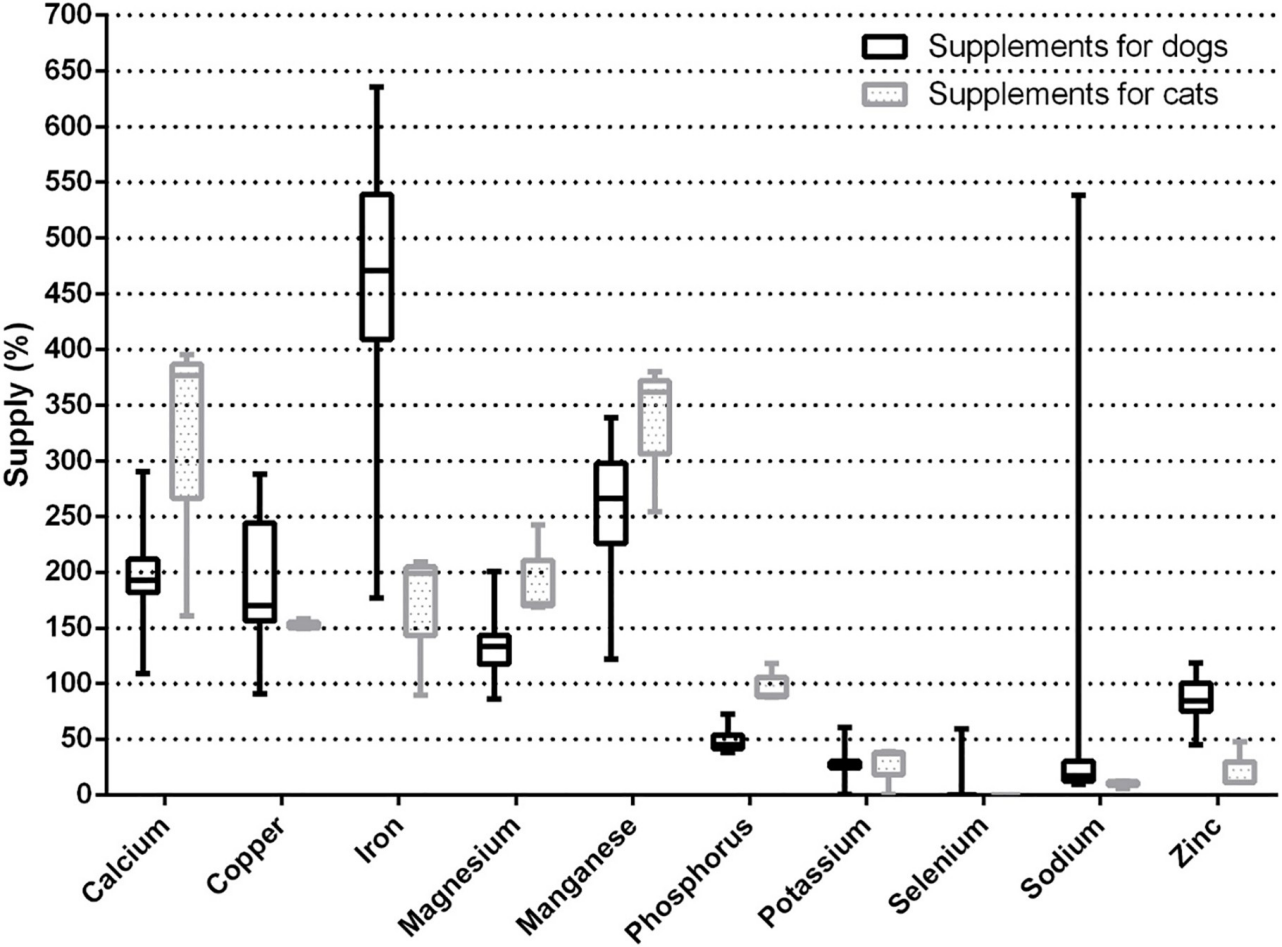
Box-and-whiskers plot of the percentages of mineral supply of the analyzed supplements according to the recommendations of NRC (2006) in mg/kg of metabolic weight (BW^0.75^ for dogs and BW^0.67^ for cats).

**Fig 2:**
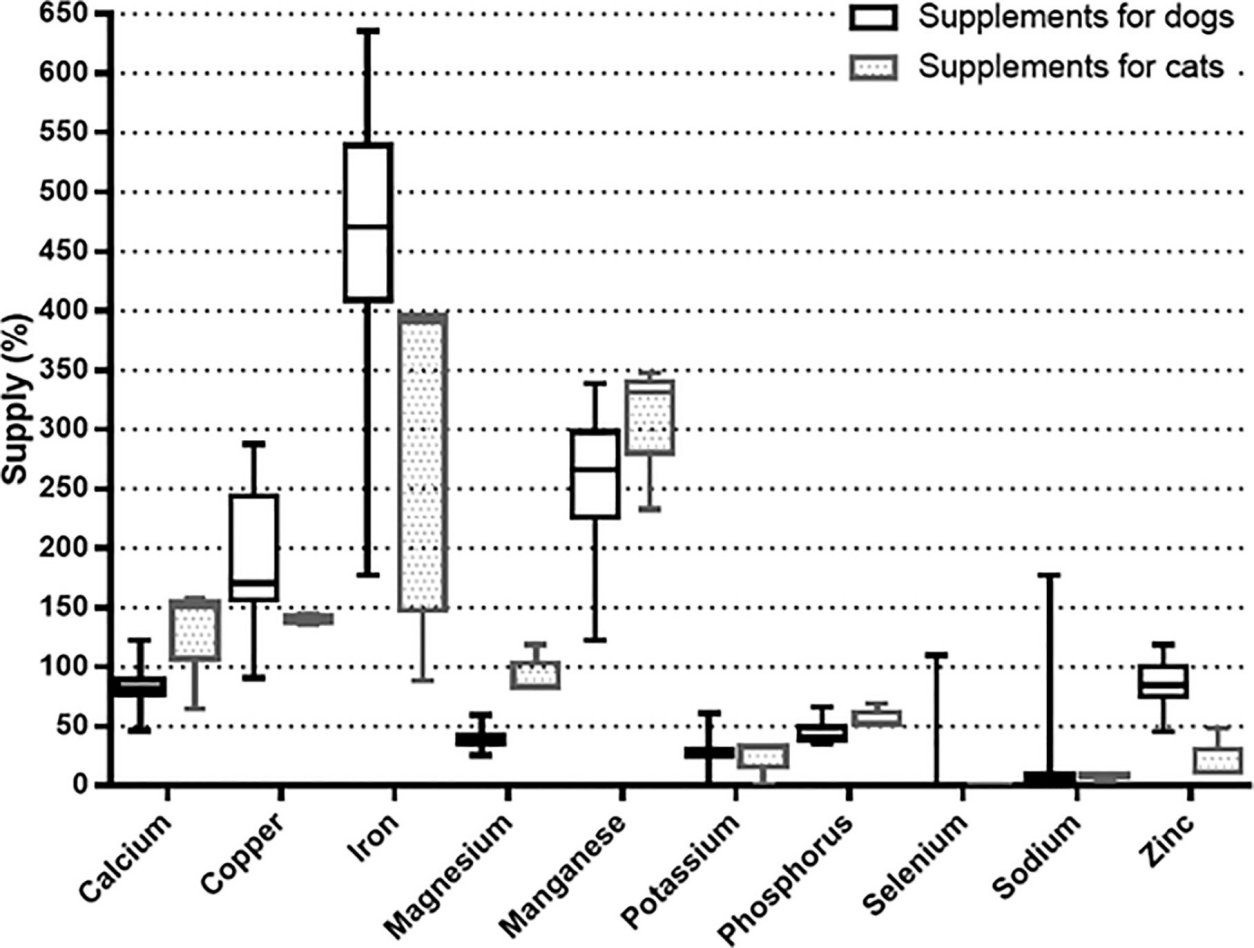
Box-and-whiskers plot of the percentages of mineral supply of the analyzed supplements according to the recommendations of FEDIAF (2020) in mg/kg of metabolic weight (BW^0.75^ for dogs and BW^0.67^ for cats).

The percentages of minerals supplied by the analyzed supplements in relation to the NRC [47] and FEDIAF [48] recommendations are illustrated in mg/kg of MW in Figs 1 and 2, respectively. Table 1 shows the results of the concentrations of essential minerals analyzed in mg/kg of natural matter in the analyzed supplements. Table 2 shows the results of the amount of toxic metals that each supplement provided per kg of BW, and Table 3 shows the concentrations of toxic metals (mg/kg DM) in the final product (homemade food) from the analyzed supplements. Regarding the supplement indicated for growing dogs, only the amount of product to be added per kg of prepared food was presented on the product’s label, so that it was only possible to estimate the amount of essential minerals that this product provides per kg of homemade food in dry matter and not the quantity per kg of MW (Table 4).

**Table 1:** Concentrations of minerals in original matter of the analyzed supplements.

	Ca (g/kg)	Cu (mg/kg)	Fe (mg/kg)	K (g/kg)	Mg (g/kg)	Mn (mg/kg)	Na (g/kg)	P (g/kg)	Se (mg/kg)	Zn (mg/kg)
**Supplement 1**	136.50	328.50	2350.00	67.50	4.10	157.00	42.25	57.50	5.60	1887.50
**Supplement 2**	12.85	362.50	3537.50	0.98	23.75	605.00	1.87	82.50	ND	1812.50
**Supplement 3**	146.00	174.00	3825.00	46.55	8.11	417.50	1.82	30.20	ND	209.50
**Supplement 4**	175.50	770.00	8500.00	52.50	12.62	725.00	1.75	75.50	ND	2825.00
**Supplement 5**	157.00	465.50	7175.00	59.50	10.57	570.00	4.16	60.50	ND	2137.50
**Supplement 6**	160.00	455.50	6450.00	48.85	11.76	670.00	2.9	63.00	ND	2375.00
**Supplement 7**	168.50	590.00	12062.50	40.55	11.77	675.00	2.03	111.00	ND	2987.50

Ca = calcium; Cu = copper; Fe = iron; K = potassium; Mg = magnesium; Mn = manganese; Na = sodium; P = phosphorus; Se = selenium; Zn = zinc; ND = value below the detection limit of 0.05 mg/kg DM.

**Table 2:** Quantities of toxic metals in mg/kg of body weight provided by each supplement for dogs and cats.

	Al	As	B	Ba	Be	Cd	Co	Cr	Hg	Ni	Pb	Sb	Sn	U	V
**Supplement 1**^**a**^	0.2100	0.0000	0.0072	0.0152	0.0000	0.0039	0.0049	0.0323	0.0391	0.0077	0.0424	0.0087	0.0204	0.2944	0.0319
**Supplement 1**^**b**^	0.1750	0.0000	0.0060	0.0126	0.0000	0.0033	0.0041	0.0269	0.0326	0.0064	0.0354	0.0073	0.0170	0.2454	0.0266
**Supplement 1**^**c**^	0.1583	0.0000	0.0054	0.0114	0.0000	0.0030	0.0037	0.0243	0.0295	0.0058	0.0320	0.0066	0.0154	0.2220	0.0241
**Supplement 1**^**d**^	0.1475	0.0000	0.0051	0.0106	0.0000	0.0028	0.0034	0.0227	0.0274	0.0054	0.0298	0.0061	0.0143	0.2068	0.0224
**Supplement 1**^**e**^	0.1333	0.0000	0.0046	0.0096	0.0000	0.0025	0.0031	0.0205	0.0248	0.0049	0.0269	0.0055	0.0130	0.1869	0.0203
Supplement 2*	0.4318	0.0000	0.0059	0.1338	0.0000	0.0050	0.0243	0.0131	0.0000	0.0081	0.0295	0.0046	0.0138	0.2600	0.0180
**Supplement 3**^**f**^	0.2604	0.0000	0.0204	0.0094	0.0000	0.0054	0.0089	0.0102	0.0636	0.0143	0.0424	0.0056	0.0207	0.3660	0.0108
**Supplement 3**^**g**^	0.2232	0.0000	0.0175	0.0081	0.0000	0.0047	0.0076	0.0088	0.0545	0.0122	0.0363	0.0048	0.0178	0.3137	0.0092
**Supplement 3**^**h**^	0.1953	0.0000	0.0153	0.0071	0.0000	0.0041	0.0067	0.0077	0.0477	0.0107	0.0318	0.0042	0.0155	0.2745	0.0081
**Supplement 3**^**i**^	0.1767	0.0000	0.0138	0.0064	0.0000	0.0037	0.0060	0.0069	0.0432	0.0097	0.0288	0.0038	0.0141	0.2484	0.0073
**Supplement 4**^**a**^	0.2525	0.0000	0.0175	0.0170	0.0011	0.0048	0.0117	0.0118	0.0405	0.0176	0.0385	0.0035	0.0175	0.3350	0.0116
**Supplement 4**^**b**^	0.2020	0.0000	0.0140	0.0136	0.0009	0.0039	0.0093	0.0095	0.0324	0.0141	0.0308	0.0028	0.0140	0.2680	0.0093
**Supplement 4**^**c**^	0.1683	0.0000	0.0117	0.0113	0.0008	0.0032	0.0078	0.0079	0.0270	0.0118	0.0257	0.0023	0.0117	0.2233	0.0077
**Supplement 4**^**d**^	0.1515	0.0000	0.0105	0.0102	0.0007	0.0029	0.0070	0.0071	0.0243	0.0106	0.0231	0.0021	0.0105	0.2010	0.0070
**Supplement 4**^**j**^	0.1515	0.0000	0.0105	0.0102	0.0007	0.0029	0.0070	0.0071	0.0243	0.0106	0.0231	0.0021	0.0105	0.2010	0.0070
**Supplement 4**^**k**^	0.1443	0.0000	0.0100	0.0097	0.0007	0.0028	0.0067	0.0068	0.0231	0.0101	0.0220	0.0020	0.0100	0.1914	0.0066
**Supplement 4**^**l**^	0.1347	0.0000	0.0093	0.0090	0.0006	0.0026	0.0062	0.0063	0.0216	0.0094	0.0205	0.0018	0.0093	0.1787	0.0062
**Supplement 5**^**a**^	0.2650	0.0000	0.0158	0.0119	0.0012	0.0043	0.0100	0.0100	0.0000	0.0158	0.0340	0.0033	0.0148	0.2975	0.0104
**Supplement 5**^**b**^	0.2120	0.0000	0.0126	0.0095	0.0010	0.0034	0.0080	0.0080	0.0000	0.0126	0.0272	0.0026	0.0118	0.2380	0.0084
**Supplement 5**^**c**^	0.1767	0.0000	0.0105	0.0079	0.0008	0.0029	0.0067	0.0067	0.0000	0.0105	0.0227	0.0022	0.0099	0.1983	0.0070
**Supplement 5**^**d**^	0.1590	0.0000	0.0095	0.0071	0.0007	0.0026	0.0060	0.0060	0.0000	0.0095	0.0204	0.0020	0.0089	0.1785	0.0063
**Supplement 5**^**j**^	0.1590	0.0000	0.0095	0.0071	0.0007	0.0026	0.0060	0.0060	0.0000	0.0095	0.0204	0.0020	0.0089	0.1785	0.0063
**Supplement 5**^**k**^	0.1514	0.0000	0.0090	0.0068	0.0007	0.0024	0.0057	0.0057	0.0000	0.0090	0.0194	0.0019	0.0084	0.1700	0.0060
**Supplement 5**^**l**^	0.1413	0.0000	0.0084	0.0063	0.0006	0.0023	0.0053	0.0053	0.0000	0.0084	0.0181	0.0018	0.0079	0.1587	0.0056
**Supplement 6**^**a**^	0.2253	0.0000	0.0154	0.0083	0.0010	0.0044	0.0096	0.0089	0.0000	0.0147	0.0335	0.0034	0.0140	0.2975	0.0097
**Supplement 6**^**b**^	0.1802	0.0000	0.0123	0.0067	0.0008	0.0035	0.0077	0.0071	0.0000	0.0118	0.0268	0.0027	0.0112	0.2380	0.00776
**Supplement 6**^**c**^	0.1502	0.0000	0.0103	0.0056	0.0007	0.0029	0.0064	0.0059	0.0000	0.0098	0.0223	0.0022	0.0093	0.1983	0.0065
**Supplement 6**^**d**^	0.1352	0.0000	0.0093	0.0050	0.0006	0.0026	0.0057	0.0053	0.0000	0.0088	0.0201	0.0020	0.0084	0.1785	0.0058
**Supplement 6**^**j**^	0.1352	0.0000	0.0093	0.0050	0.0006	0.0026	0.0057	0.0053	0.0000	0.0088	0.0201	0.0020	0.0084	0.1785	0.00582
**Supplement 6**^**k**^	0.1287	0.0000	0.0088	0.0048	0.0006	0.0025	0.0055	0.0051	0.0000	0.0084	0.0191	0.0019	0.0080	0.1700	0.0055
**Supplement 6**^**l**^	0.1201	0.0000	0.0082	0.0044	0.0005	0.0023	0.0051	0.0047	0.0000	0.0079	0.0179	0.0018	0.0075	0.1587	0.0052

Al = aluminum; As = arsenic; B = boron; Ba = barium; Be = beryllium; Cd = cadmium; Co = cobalt; Cr = chromium; Hg = mercury; Ni = nickel; Pb = lead; Sb = antimony; Sn = tin; U = uranium; V = vanadium

^a^ = recommendation for dogs of 5 kg

^b^ = recommendation for dogs of 10 kg

^c^ = recommendation for dogs of 15 kg

^d^ = recommendation for dogs of 20 kg

^e^ = recommendation for dogs of 30 kg

^f^ = recommendation for cats of 2.5 kg

^g^ = recommendation for cats of 3.5 kg

^h^ = recommendation for cats of 5 kg

^i^ = recommendation for cats of 7 kg

^j^ = recommendation for dogs of 25 kg

^k^ = recommendation for dogs of 35 kg

^l^ = recommendation for dogs of 45 kg

* = recommendation per kg of body weight, regardless of size.

**Table 3:** Concentrations of toxic metals from the supplements analyzed in the final product (homemade food) in mg/kg of dry matter.

	Al	As	B	Ba	Be	Cd	Co	Cr	Hg	Ni	Pb	Sb	Sn	U	V
**FDA (2011)**	200*	12.5*	15*	10*	5*	10*	2.5*	10*	0.27*	50*	10*	40*	10*	10*	200*
**Supplement 1**	2.5000	0.0000	0.0860	0.1805	0.0000	0.0469	0.0580	0.3840	0.465	0.0920	0.5050	0.1040	0.2430	3.5050	0.3805
**Supplement 2**	6.9080	0.0000	0.0940	2.1400	0.0000	0.0800	0.3888	0.2096	0.0000	0.1288	0.4720	0.0728	0.2212	4.1600	0.2884
**Supplement 3**	2.6040	0.0000	0.2036	0.0944	0.0002	0.0544	0.0888	0.1024	0.636	0.1428	0.4240	0.056	0.2072	3.6600	0.108
**Supplement 4**	2.0200	0.0000	0.1402	0.1356	0.0092	0.0386	0.0932	0.0946	0.324	0.1410	0.3080	0.0276	0.1400	2.6800	0.0928
**Supplement 5**	2.1200	0.0000	0.1260	0.0948	0.0096	0.0342	0.0800	0.0800	0.0000	0.1264	0.2720	0.0264	0.1182	2.3800	0.0836
**Supplement 6**	1.8020	0.0000	0.1234	0.0666	0.0079	0.0350	0.0766	0.0710	0.0000	0.1178	0.2680	0.0268	0.1120	2.3800	0.0776
**Supplement 7**	10.7400	0.0000	0.5112	0.2442	0.0690	0.1242	0.3444	0.2952	0.0000	0.5916	0.9720	0.0792	0.4080	9.1800	0.3588

Al = aluminum; As = arsenic; B = boron; Ba = barium; Be = beryllium; Cd = cadmium; Co = cobalt; Cr = chromium; Hg = mercury; Ni = nickel; Pb = lead; Sb = antimony; Sn = tin; U = uranium; V = vanadium

* = maximum tolerated level (MTL) recommended by the FDA (2011) for toxic metals in mg/kg of dry matter. The MTL of the elements: B, Ba and Sn did not have their values recommended by the FDA (2011), therefore they were extrapolated from mammals known to be more sensitive and the value was divided by 10, as a safety factor.

**Table 4:** Estimate of the concentrations in mg/kg of dry matter of the minerals in the final product (homemade food) from the vitamin-mineral supplement for growing dogs.

	Ca (g/kg)	Cu (mg/kg)	Fe (mg/kg)	K (g/kg)	Mg (g/kg)	Mn (mg/kg)	Na (g/kg)	P (g/kg)	Se (mg/kg)	Zn (mg/kg)
**FEDIAF (2020)**	**8.0**^**1**^	**11.0000**^**1**^	**88.0000**^**1**^	**4.4**^**1**^	**0.4**^**1**^	**5.6000**^**1**^	**2.2**^**1**^	**7.0**^**1**^	**0.4000**^**1**^	**100.0000**^**1**^
**NRC (2006)**	**8.0**^**2**^	**11.0000**^**3**^	**72.0000**^**2**^	**4.4**^**3**^	**0.18**^**2**^	**5.6000**^**3**^	**2.2**^**3**^	**10.0**^**3**^	**0.2100**^**2**^	**40.0000**^**2**^
**Supplement 7**	1.26375	4.4250	90.4688	0.304125	0.0883125	5.0625	0.0151875	0.8325	0.000	22.4063

^1^ = minimum recommendation of FEDAF (2019) (in mg / kg DM) for growing dogs under 14 weeks

^2^ = minimum recommendation of NRC (2006) (in mg / kg DM) for growing dogs above 14 weeks

^3^ recommended allowance of NRC (2006) (in mg / kg DM) for growing puppies after weaning; Ca = calcium; Cu; copper; Fe = iron; K = potassium; Mg = magnesium; Mn = manganese; Na = sodium; P = phosphorus; Se = selenium; Zn = zinc.

According to the recommendations on the supplement labels and the results obtained in the laboratory, it was observed that most of the products did not guarantee the recommendations (per mg/kg MW) of FEDIAF [48] for the following minerals: calcium (Ca), phosphorus (P), magnesium (Mg), potassium (K), selenium (Se), sodium (Na), and zinc (Zn); and of NRC [47] for the same minerals, except Ca and Mg. As for Supplement 7, it did not provide the FEDIAF [48] recommended levels in mg/kg DM in the final product (homemade food) for the following elements: Ca, copper (Cu), K, Mg, Mn, Na, P, Se, and Zn; and in relation to the recommendations of the NRC [47], it did not meet the requirements of the same minerals mentioned above. Regarding the amount of toxic metals supplied per kg of BW, three supplements provided more than 0.02 mg of mercury/kgBW, which is the safe upper limit used to establish the maximum tolerated level of this element. All other supplements did not result in concentrations of any element above the MTLs in mg/kg DM of the unconventional food. Only one supplement presented selenium concentrations above the detection limits of 0.05mg/kg DM.

## Discussion

The main findings of this study are that the majority of the analyzed supplements, if used in the quantities recommended by the manufacturers, are not guaranteed to supply the minimum recommendations of FEDIAF [48] for Ca, K, Mg, Na, P, Se, and Zn; as well as the NRC [47] recommendations for K, Mg, Na, P, Se, and Zn. In addition, only one supplement had detectable concentrations of selenium. Regarding the toxic metal concentrations, the main findings were high concentrations of Hg in three supplements, through which the MTL of this element would be exceeded in the estimate of mg Hg/kg DM. Furthermore, these supplements presented concentrations of mercury that would exceed the safe upper limit observed by Charbonneau et al. [51] if used as recommended on the product label.

In the formulation of unconventional diets, the use of vitamin-mineral supplementation is a mandatory practice, according to Parr and Remillard [6], as meeting the requirements of these nutrients cannot be achieved only with the use of common ingredients. According to Pedrinelli et al. [4], this type of food must be formulated by trained professionals, who know the nutritional requirement of dogs and cats, as well as the nutritional composition of the different ingredients used in the formulation. The findings of the present study reinforce this, since if this type of food is prepared by professionals with little knowledge about nutrition, even if supplementation is used according to the manufacturers’ recommendations, the foods may present nutritional deficiencies. A study conducted by Pedrinelli et al. [4] also reinforces this. The authors analyzed 100 homemade food recipes, 75 for dogs and 25 for cats, and 20% of the recipes for dogs had the addition of vitamin-mineral supplements. The diets that did not include vitamin-mineral supplements presented no difference in Ca, P, Ca: P ratio, K, Mg, and Na concentrations when compared to diets that contained supplements. This demonstrates that if unconventional foods are not formulated by professionals who are knowledgeable about the composition of ingredients and nutritional requirements of pets, even with the inclusion of vitamin-mineral supplements, diets may be deficient. This justifies the fact that the World Small Animal Veterinary Association considers the use of unconventional foods as a risk factor for the development of nutritional deficiencies [5].

In total, 5/6 VMS for dogs did not guarantee the minimum Ca recommendation of FEDIAF [48]. Low Ca intake can result in secondary nutritional hyperparathyroidism (SNH) [52], an alteration that has been quite common in the past, due to the unbalanced homemade food that was provided to pets [53]. Nowadays, SNH is rare due to the use of complete and balanced commercial foods which supply the animals’ calcium requirements. SNH can result in rubber jaw syndrome, a set of lesions characterized by loose teeth and osteopenia of the skull bones [53]. In addition, SNH implies hypocalcemia, which can cause muscle spasms, excitation, convulsion and osteopenia, which causes bone fractures [52]. In the literature, there are two case reports of puppies fed a calcium-deficient homemade diet that showed bone alterations [54, 55]. In the case conducted by Hutchinson et al. [54], a sexually intact male Saint Bernard puppy was fed a homemade diet from the 11th week of life until 8 months of age, which supplied only 39.5% of the minimum calcium recommendation of NRC [47] and had a calcium:phosphorus ratio of 1:1.43. After a few months, the animal presented bilateral lameness of the forelimbs, and the radiographic findings were consistent with bilateral osteochondritis dissecans of the shoulder joints. In addition, diffuse osteopenia was observed on radiographs of the mandible and long bones, which revealed bone demineralization. In the serum biochemical analysis, severe hypocalcemia and hyperphosphatemia were observed.

In the case report by Tal et al. [55], lameness and pain in pelvic limbs was reported in a 6-month-old Giant Schnauzer puppy that was fed a homemade diet since the two months of age. The diet followed an internet recipe, which supplied only 7.5% of the calcium recommendation of NRC [47], and had a calcium:phosphorus ratio of 1:4 and vitamin D concentration below the recommendations. In laboratory tests, ionized calcium and 25-hydroxivitamin D_3_ [25(OH)D_3_] were below the minimum reference range and, although the serum PTH concentration was within the reference range for the species, authors suspected the presence of SNH, along with rickets.

These studies reinforce the importance of vitamin-mineral supplements with adequate calcium concentrations to guarantee the minimum recommendations, because most of the common ingredients have very low calcium concentrations and inadequate calcium:phosphorus ratio. Even in studies that analyzed the nutritional adequacy of homemade diet recipes published in books and websites, calcium was one of the nutrients most frequently below the recommendations of FEDIAF or NRC, ranging from 35.0 to 82.7% of the recipes for dogs with insufficient calcium concentrations [4, 8, 9] and 37.2 to 73.1% of recipes for cats [4, 8, 10]. It is worth mentioning that 5/6 VMS indicated for dogs and analyzed in the present study did not guarantee the minimum calcium recommendation of FEDIAF [48]. In addition, Supplement 2 presented calcium:phosphorus ratio of 1:6.42, which can further compromise the homemade diets inadequacies.

Regarding selenium, only 1/7 VMS showed detectable concentrations of this mineral. Regarding selenium dietary deficiency, few studies have evaluated this issue. In a study conducted by Van Vleet [56], selenium deficiency was induced in puppies and, after 6 to 8 weeks, clinical changes were observed, such as anorexia, dyspnea, and coma. In addition, histopathological changes such as muscle degeneration and renal mineralization were observed. In studies that analyzed the nutritional adequacy of homemade diet recipes for dogs and cats, a considerable portion of that recipes (19.1–56.1%) showed selenium concentrations below the recommendations of FEDIAF or NRC [4, 8–10], which demonstrates the importance of vitamin-mineral supplements providing adequate quantities of this mineral.

No VMS for dogs and only one for cats guaranteed the minimum magnesium recommendation of FEDIAF [48]. It has also been reported that a high percentage of homemade diet recipes have magnesium concentrations below FEDIAF or NRC recommendations, ranging from 26.7 to 57.3% of recipes for dogs [4, 8, 9] and 4.0 to 23.1% of recipes for cats [4, 8, 10]. Magnesium deficiency has been reported in diets without added magnesium for growing dogs, which resulted in hyperextension of the carpal joints and paralysis of the hind limbs [57]. In a subsequent study conducted by Bunce et al. [58], growing dogs fed a diet with magnesium concentrations below 140mg/kg DM developed anorexia, weight loss, hyperextension of the carpal joints, and posterior ataxia within 3 to 6 weeks after feeding. In the post-mortem histopathological evaluation, the authors observed mineralization of the thoracic aorta in animals that presented clinical evidence of magnesium deficiency. In another study, conducted by Stahlman et al. [59], clinical signs of magnesium deficiency, such as lameness and carpal hyperextension, were observed in growing Beagles after 4 weeks of feeding a diet that contained 136mg of Mg/kg DM. In addition, hypomagnesaemia is associated with increased vascular reactivity [60] and low circulating concentrations of magnesium results in increased vascular tone, which can exacerbate the high afterload state of congestive heart failure in dogs, resulting in more arrhythmias [61]. These effects of magnesium on vascular tone are related to its role in the movement of calcium since this mineral can act as an antiarrhythmic agent by limiting intracellular calcium overload and supporting intracellular potassium replacement, and is therefore important in the correction of hypokalemia [62].

Regarding zinc, most of the analyzed VMS did not meet the minimum recommendations of FEDIAF and NRC for this mineral. It is worth mentioning that zinc is one of the most frequently deficient nutrients in homemade diet recipes, with percentages of recipes for dogs with zinc concentrations below FEDIAF or NRC recommendations ranging from 66.7 to 78.7% [4, 8, 9], and in recipes for cats, the percentage of diets with insufficient zinc concentrations varied from 61.7 to 88.5% [4, 8, 10]. Therefore, it is important that the VMS supply adequate zinc quantities in order to reduce the risk of insufficient zinc intake. Concerning dietary zinc deficiency, alopecia and mucocutaneous junction lesions can be observed, in addition to parakeratosis in the histological evaluation [63]. In cats, abnormalities in spermatogenesis have been reported in male cats who consumed a diet containing 15mg/kg DM of zinc for eight months [64].

No analyzed VMS guaranteed the minimum potassium recommendation of FEDIAF [48] and NRC [47], for both dogs and cats. This can be worrying, because potassium is below FEDIAF and NRC recommendations in a considerable percentage of homemade diet recipes as previously published, ranging from 7.5 to 94.7% of recipes for dogs [4, 8, 9] and 33.0 to 96.0% of recipes for cats [4, 8, 10]. As for potassium deficiency, it has been observed that it can influence blood pressure and renal perfusion in adult dogs in the short term, but without resulting in clinical signs [65]. In contrast, Dow et al. [66] observed that when cats consumed a diet with 3.4g of K/kg DM, increased serum creatinine and potassium excretions were observed, characteristic signs of kidney disease. Regarding the dietary deficiency of phosphorus, Kienzle et al. [67] observed clinical signs in adult cats fed diets with a Ca: P ratio of 4:1. The authors observed hemolytic anemia, locomotion disorders, and metabolic acidosis.

Another important fact that makes the guarantee of minimum mineral recommendations by VMS even more important is that a considerable portion of the owners do not correctly follow the instructions of the recipes, as already demonstrated in some studies [3, 68, 69], which results in a higher risk for nutritional deficiency. In the study conducted by Johnson et al. [68], it was observed that only 13.0% of the owners who exclusively provided homemade diets for their dogs followed the instructions provided. Oliveira et al. [69] reported that 50.0% of the owners who provided a homemade diet for dogs or cats did not follow the instructions correctly, only 15.2% of the interviewed owners had a scale to weigh the ingredients, and 28.3% admitted not using the prescribed supplementation. In the study conducted by Halfen et al. [3], 110 dog owners who provided homemade diets for their pets were interviewed, and it was observed that 60.0% of themchanged the prescribed recipe, such as altering the type of meat or including another ingredient, and 35.1% of the owners admitted that they did not correctly follow the prescribed amount of each ingredient. This demonstrates that even if a homemade diet is formulated by a trained professional, it can become unbalanced and deficient in nutrients due to the changes constantly made by the owners.

Regarding the intake of Hg above MTL, Charbonneau et al. [51] observed that cats that consumed amounts higher than 0.02mg of Hg/kg BW presented ataxia, loss of balance, and motor incoordination. In the histopathological exam, loss of nerve cells with replacement by reactive and fibrillar gliosis after 14 to 40 weeks of treatment was observed. It is worth mentioning that in our study, three supplements provided more than 0.02mg Hg/kg BW in the amount recommended by the manufacturer and, if homemade food is prepared with ingredients contaminated with Hg, the intake of this element can be even higher. These results suggest that such supplements may imply health risks for dogs and cats concerning mercury poisoning.

In dogs, a case of acute mercury poisoning from ingesting a mercury barometer has been reported [70]. The authors have demonstrated that acute mercury poisoning can cause a series of clinical signs, such as vomiting, bloody diarrhea, leukocytosis, splenomegaly, hepatomegaly, nephromegaly, uremia, hypoproteinemia, metabolic acidosis, proteinuria, and glycosuria. In the post-mortem histopathological exam, kidney lesions consistent with severe and diffuse necrosis of the proximal tubular epithelium with multifocal mineralization were detected. In the assessment of liver tissue, moderate multifocal hepatocellular necrosis was observed. In another case report described by Farrar et al. [71], a dog presented progressive neurological signs (bilaterally absent pupillary light reflexes and horizontal nystagmus, weak withdrawal reflexes in all limbs, opisthotonos, and tetraparesis) and gastrointestinal signs (vomiting and bloody diarrhea), in addition to contamination of peripheral blood in the cerebrospinal fluid and lymphocytopenia. In the post-mortem evaluation, microlesions were observed in the brain (generalized hypertrophy and proliferation of perivascular adventitial cells), spinal cord (severe axonal swelling and degeneration with occasional spheroid formation, moderate edema, and myelin digestion chambers), kidneys (tubular cell degeneration and necrosis with proteinaceous cast formation), and intestines (acute, diffuse, superficial villous necrosis of the duodenum, jejunum, and ileum). Due to clinical signs, heavy metal intoxication was suspected and, when liver and kidney analysis were performed, high hepatic and renal mercury concentrations were observed (2.8 and 3.3ppm wet weight basis, respectively; reference range: < 0.1ppm). Therefore, the authors associated the changes with mercury poisoning, since it has been reported in other mammalian species that mercury poisoning can cause anorexia, hypothermia, nervous dysfunction, renal dysfunction, gastrointestinal signs, shock, and death [72–76].

Mercury contamination in VMS could be associated with some factors, such as soil contamination, which is the source of the extraction of various components of the VMS evaluated in the present study, and to the contamination of animal products that are often used in VMS as flavoring agents. In recent years, an increase in environmental mercury concentrations has been observed [77]. This increase can be justified by the intensification of human activities, such as deforestation, oil extraction, road construction and mining, which increase the extraction or solubilization of mercury from the systems [78, 79].

It is known that a large mercury amount is transported by air masses, or by waterways over long distances from industrial and populated areas [77, 80]. This long-range atmospheric transport may also be responsible for the mercury accumulation in ingredients present in composition of the VMS analyzed in the present study. In addition, recent studies have observed the impact of this mercury transport on species from remote locations [81–83], which further justifies the hypothesis that in recent years, the combination of industrial activities and atmospheric transport has resulted in an increase in concentrations of mercury in soils [84]. According to Liu et al. [84], with the increase in production and industrial use since the 19th century, pollution by mercury has intensified. Human activity after the industrialization process increased the mercury content in soil and sediments, which makes mining and industrial production activities the main reasons for soil pollution by mercury.

With regard to the animal origin products contamination, an important factor to be considered is the biomagnification process, a process of absorption and accumulation of chemical substances or compounds in the body. It is well known that methylmercury, in the aquatic environment, undergoes bioaccumulation and biomagnification along the food chain, particularly in aquatic animals [85, 86]. Therefore, if these aquatic species are used as ingredients in pet food, they can contribute to mercury contamination in the final product, as is the case of vitamin-mineral supplements, in which fish by-products are often used as flavoring agents.

The present study had as limitations the non-analysis of vitamins in the vitamin-mineral supplements evaluated. The study evidences that the majority of vitamin-mineral supplements analyzed do not guarantee the minimum recommendations for most essential minerals, even when the quantity recommended by the manufacturer was considered. Therefore, if unconventional foods are not formulated by trained professionals, they may have deficiencies in some minerals. In addition, three of the seven supplements analyzed may imply risks of Hg poisoning in dogs and cats that consume them.
